# Implications of Transcranial Magnetic Stimulation as a Treatment Modality for Tinnitus

**DOI:** 10.3390/jcm10225422

**Published:** 2021-11-20

**Authors:** Alexa J. Denton, Ariel Finberg, Peter E. Ashman, Nathalie B. Bencie, Tricia Scaglione, Brianna Kuzbyt, Fred F. Telischi, Rahul Mittal, Adrien A. Eshraghi

**Affiliations:** 1 University of Miami Ear Institute, Department of Otolaryngology, University of Miami Miller School of Medicine, Miami, FL 33136, USA; adent003@med.fiu.edu (A.J.D.); afinberg@med.miami.edu (A.F.); pea31@njms.rutgers.edu (P.E.A.); nbencie@purdue.edu (N.B.B.); tscaglione@med.miami.edu (T.S.); bkuzbyt@med.miami.edu (B.K.); FTelischi@med.miami.edu (F.F.T.); r.mittal11@med.miami.edu (R.M.); 2 Department of Neurological Surgery, University of Miami Miller School of Medicine, Miami, FL 333136, USA; 3 Department of Biomedical Engineering, University of Miami, Coral Gables, FL 33146, USA; 4 Department of Pediatrics, University of Miami Miller School of Medicine, Miami, FL 33136, USA

**Keywords:** TMS, tinnitus, neuromodulation, auditory cortex, synaptic plasticity, non-invasive

## Abstract

Repetitive transcranial magnetic stimulation (rTMS) is a non-invasive, neuromodulating technique for brain hyperexcitability disorders. The objective of this paper is to discuss the mechanism of action of rTMS as well as to investigate the literature involving the application of rTMS in the treatment of tinnitus. The reviewed aspects of the protocols included baseline evaluation, the total number of sessions, frequency and the total number of stimuli, the location of treatment, and the outcome measures. Even with heterogeneous protocols, most studies utilized validated tinnitus questionnaires as baseline and outcome measures. Low frequency (1 Hz) stimulation throughout 10 consecutive sessions was the most widely used frequency and treatment duration; however, there was no consensus on the total number of stimuli necessary to achieve significant results. The auditory cortex (AC) was the most targeted location, with most studies supporting changes in neural activity with multi-site stimulation to areas in the frontal cortex (FC), particularly the dorsolateral prefrontal cortex (DLPFC). The overall efficacy across most of the reviewed trials reveals positive statistically significant results. Though rTMS has proven to impact neuroplasticity at the microscopic and clinical level, further studies are warranted to demonstrate and support the clinical use of rTMS in tinnitus treatment with a standardized protocol.

## 1. Introduction

Repetitive magnetic transcranial stimulation (rTMS) is a non-invasive neuromodulation modality that has been utilized within the neurological and psychiatric communities [1,2,3,4]. Since its development as a therapeutic tool in 1985 [5], rTMS has been shown to provide various degrees of symptomatic relief for conditions such as depressive disorders, pain, aphasia, movement disorders, motor stroke, multiple sclerosis, epilepsy, disorders of consciousness, Alzheimer’s disease, schizophrenia, substance abuse, and addiction [6,7,8,9]. Considering the beneficial effects, there has been an emerging interest in utilizing rTMS for auditory disorders such as tinnitus [10,11,12,13].

Tinnitus is the perception of sound in the absence of an external auditory stimulus [14]. It can be caused by several different underlying conditions that affect a wide range of structures between the ear and the brain itself, leading to variability in clinical manifestations. It has been suggested that damage to structures such as the auditory nerve or hair cells within the cochlea can lead to changes in plasticity that enhance the activity in the auditory cortex (AC), as well as other non-auditory areas of the brain, leading to this perceived sound [15,16]. There are also ototoxic medications such as aspirin, cisplatin, aminoglycosides, and loop diuretics that have been associated with tinnitus [17,18]. It is known that around 10–15% of the United States population has reported experiencing tinnitus to varying degrees [19], with many reporting a significant impact on their quality of life. With many individuals affected by this debilitating condition, there have been various treatments utilized in an attempt to ameliorate tinnitus symptoms. These treatments include pharmacotherapies (e.g., antidepressants), ear-level devices (e.g., hearing aids), sound generators, behavioral therapy, and even cochlear implants [20,21]. Among the most recent treatment methods is the utilization of non-invasive techniques that focus on the electrical or magnetic stimulation of specific brain regions that are known to be associated with tinnitus [14]. Though a newer therapy, many studies have examined the utilization of rTMS as a novel therapeutic tool for tinnitus [10,11,12,13].

In this scoping review article, we will first examine the proposed mechanisms by which rTMS modulates neural connections. We will then discuss the most recent clinical trials and meta-analyses as well as potential roadblocks with rTMS in order to generate potential further steps that can be taken to include rTMS as a future treatment modality for tinnitus.

## 2. Background on rTMS and Tinnitus

### 2.1. Technology Overview of rTMS 

**Figure 1:**
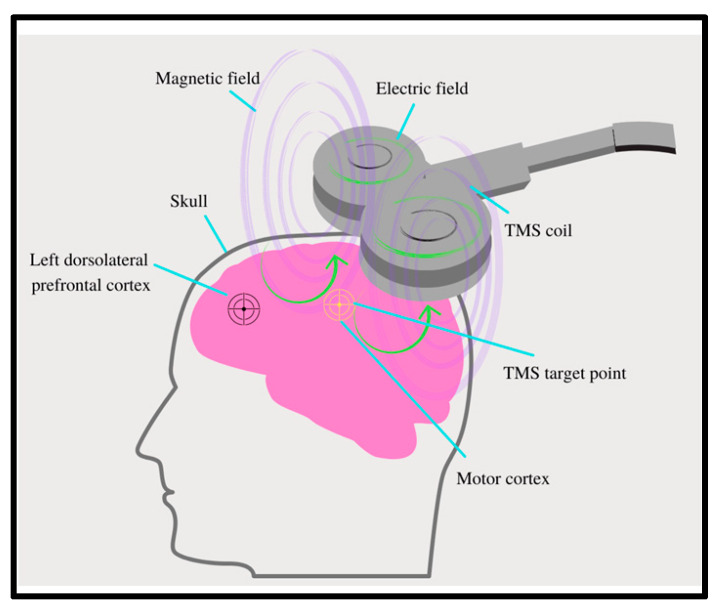
Components of repetitive magnetic transcranial stimulation (rTMS): rTMS is a non-invasive therapy that utilizes a wire coil connected to a magnetic stimulator. The electromagnetic current generated by the coil is applied to the scalp of patients directed at the area of interest, modulating neuronal excitability. The dorsolateral prefrontal cortex (DLPFC) is a common target of rTMS utilization in tinnitus.

The therapy provided by rTMS is non-invasive and delivered through the use of a wire coil connected to a magnetic stimulator that generates an electromagnetic current [9] (Figure 1). This electromagnetic field is then applied closely to the scalp of patients at the location of interest with multiple pulses, ultimately modulating the excitability of the neurons within the cortex (Figure 1). The pulses generated by this magnetic field can be either excitatory, with a frequency greater than 5 hertz (Hz), or inhibitory, with a lower frequency, that is usually ≤1 Hz. The application of either frequency depends on the specific treatment goals [9].

**Figure 2:**
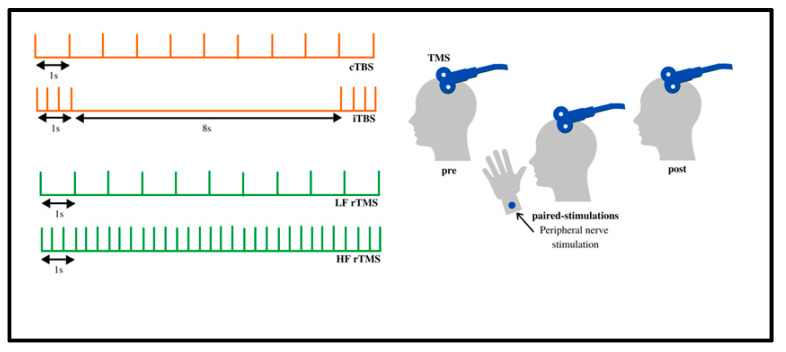
Continuous, repetitive, and intermittent TMS: The various protocols can be classified into continuous theta burst stimulation (cTBS) and intermittent theta burst stimulation (iTBS). In cTBS, three pulses are given at 50 Hz with an inner frequency of 5 Hz for either 20 or 40 s. iTBS consists of 20 bursts every 2 s at 0.1 Hz. iTBS is considered to be excitatory, while cTBS is considered to be inhibitory. Adapted from Klomjai et al. [24].

The varying frequencies of rTMS can be subsequently stratified into more precise protocols for a therapeutic use called theta burst stimulation (TBS). Further classification leads to either continuous theta burst stimulation (cTBS) or intermittent theta burst stimulation (iTBS), each applied with varying frequencies and time frames. In cTBS, three pulses are given at 50 Hz, with a 5 Hz inter-burst pulse delivered for either 20 or 40 s [22]. iTBS consists of 20 bursts every 2 s at 0.1 Hz (Figure 2). The difference between these two modalities is that iTBS produces an excitatory response, while cTBS produces an inhibitory response [23].

Whether excitatory or inhibitory, the stimulation generated by rTMS ultimately induces a depolarization within the cell membrane of neurons. This depolarization results in an alteration in neuronal connections called synaptic plasticity, which can last beyond the actual therapy for an uncertain period of time [25,26]. The resultant synaptic plasticity can be attributed to the long-term depression (LTD) or long-term potentiation (LTP) between existing synapses elicited by the inhibitory or excitatory frequencies generated by rTMS, respectively [23].

LTP leads to the amplification of certain neuronal connections, while LTD weakens such connections [27]. In a clinical trial setting, this variation in synaptic plasticity is measured by the motor-evoked potentials (MEPs), correlating with whether or not the proposed stimulus elicited the expected excitatory or inhibitory response. MEPs are usually measured by the intrinsic hand muscle movement, or the lack thereof, which correlates to the stimulated cortical region of interest [28]. The MEPs for iTBS have been described to be elevated, while the MEPs for cTBS are dampened, defining the excitatory and inhibitory effects of rTMS [23].

### 2.2. Proposed Mechanism of Action

**Figure 3:**
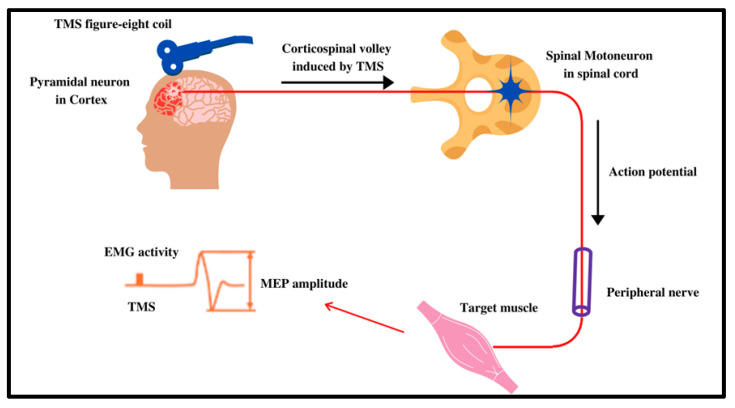
Mechanism of action of TMS: To quantify the strength and confirm the activity of the TMS, MEPs are used. Once the TMS coil is fired, cortical neurons signal via the corticospinal tract to the stimulated targeted muscle activity. These MEPs can be quantified as excitatory or inhibitory depending on the muscle’s movement or lack thereof. Adapted from Klomjai et al. [24].

The mechanism of action of rTMS has been widely studied since it was first theorized to be used for the management of neurological and psychiatric disorders; however, it is still not completely understood. As previously mentioned, the target activity of rTMS is its ultimate effect on synaptic plasticity and neural circuits (Figure 3).

Peng et al. [29] used both animal and biological models to evaluate the variations in gene and protein expression resulting from rTMS. Recently, a study by Thomson et al. [30] was successful in using human-like neuron models to examine similar in vitro parameters of the resulting plasticity. Imaging with various modalities, such as functional magnetic resonance imaging (fMRI) and positron emission tomography (PET), has also been introduced to visually analyze the effect rTMS has on the neuronal networks during treatment [31].

At a molecular level, findings from studies that utilized animal models have shown that rTMS with excitatory frequencies alters the expression of both the *N*-methyl-*D*-aspartate (NMDA) receptor and the brain-derived neurotrophic factor (BDNF) genes and proteins, which are excitatory neurotransmitters. This supports the idea that rTMS plays a role in altering neuronal plasticity based on gene expression [26,29,30]. Additionally, the release of intracellular calcium stores has been observed, further supporting this hypothesis [30]. Other genes found to be affected in rat models include C-FOS, a marker for excitation in cells, and Early Growth Response 1 (EGR1), which is postulated to be a marker for the induction of LTD and LTP [30]. Additionally, increased gamma-aminobutyric acid (GABA) neurotransmission has been implicated as a principal change associated with inhibitory stimulation [26]. To examine the effects on a larger scale, Noh et al. [26] investigated the effect that inhibitory cTBS has on cortical oscillations between varying regions of the brain. They found that a decrease in low beta brain rhythms was observed shortly after cTBS stimulation, demonstrating a decrease in interhemispheric connectivity [26]. There is also evidence that suggests that excitatory stimulation regulates inhibitory interneurons, leading to a dampening effect on neural activity on target cortical regions [29].

A study by Thomson et al. [30] sought to reproduce the results of previous investigations by examining the variations in the BDNF-TrkB (Tropomyosin receptor kinase B) gene expression in animal models through utilizing SH-SY5Y neuroblastoma cells as a human-like neuron model. Following iTBS sessions, the following genes within the BDNF-TrkB pathway were analyzed: Mitogen-Activated Protein Kinase 9 (MAPK9), Neurotrophic Regulator Tyrosine Kinase 2 (NTRK2), B-cell lymphoma 2 (BCL2), Tubulin Beta Class III (TUBB3), cAMP Responsive Element Binding Protein 1 (CREB1), and EGR1. The results of this study demonstrated an increased expression of NTRK2, MAPK9, and BCL2 after 24 h, supporting previous evidence of increased BDNF expression and therefore synaptic plasticity. EGR1 was transiently elevated within 10 min to 2 h of stimulation, supporting its role in initiating plasticity [30].

A limitation with animal and human-like neuron models is the lack of visualization of how these treatments impact the intact human brain in real-time. With the propulsion of rTMS into clinical medicine as a promising treatment for a multitude of neurological conditions, studies have developed the technology itself to be used along with fMRI and PET to view the activity of targeted locations of the brain [31]. Utilizing imaging before and after treatment may give more insight into the areas activated by rTMS that are not well known. A study was successful in revealing “propagation pathways,” as well as detecting activation in distant cortical locations beyond the stimulation site [31]. With the addition of fMRI and PET to the protocol, preset parameters of treatment can be finely manipulated to achieve the desired location of activity in an individualized manner. The current literature on this topic describes multiple application designs, each with its own set of advantages and disadvantages. Further research is warranted to bridge the gap between the experimental and clinical use of rTMS and imaging modalities.

### 2.3. Pathophysiology of Tinnitus

The causes of tinnitus, though variable, ultimately affect structures within the ear and brain associated with the AC, leading to the perception of sound without an actual stimulus. The most common cause of tinnitus is related to the loss of peripheral hearing; however, many patients with tinnitus present with normal hearing sensitivity. This suggests the involvement of non-auditory centers of the brain [32].

There are various hypotheses that describe the mechanism of action of tinnitus in the presence of hearing loss. A decreased sensorineural hearing input due to damage to cochlear hair cells and/or the auditory nerve leads to the downregulation of GABA inhibition This inhibition can subsequently lead to increased neural activity in other structures involved in the auditory pathway that are functionally unimpaired [33].

While there is agreement that the AC in the temporal lobe plays a role in the perception of tinnitus, a study [34] identified other possible non-auditory locations within the brain that have been implicated in its pathogenesis. These alternative areas are characterized by their roles in factors beyond actual noise perception such as attention direction, salience attribution, emotional processing, and memory function [32]. The dorsomedial prefrontal cortex (DMPFC) and anterior cingulate cortex (ACC) are responsible for the individual’s cognizance of tinnitus. Other central structures such as the amygdala, anterior insula, and hippocampus play a role in the manifestation of agitation, anxiety, and emotional stress related to tinnitus [32].

Some of these structures have been associated with increased activity and neural plasticity resulting in tinnitus pathogenesis, and have thus become the targets of neuromodulation treatment in various rTMS trials. In the remainder of this paper, we review and discuss studies that have targeted these various locations and outline the most up-to-date conclusions on rTMS as a treatment modality for tinnitus.

### 2.4. Questionnaires for Evaluating Tinnitus 

Several validated questionnaires are used clinically to assess the nature and impact of tinnitus. The Tinnitus Handicap Inventory (THI), Tinnitus Questionnaire (TQ), Tinnitus Severity Scale (TSS), and Visual Analog Scale (VAS), which are described below, were used alone or in varying combinations in the studies we examined. Other scales have been developed that were not used in any of the studies reviewed; these include: the Tinnitus Handicap Questionnaire, the Subjective Tinnitus Severity Scale, Tinnitus Reaction Questionnaire, Tinnitus Severity Grading, Tinnitus Severity Index, and the Intake Interview for Tinnitus Retraining Therapy [35].

The THI is a 25-question survey where each question can be answered with “yes, no, or sometimes”, with each response counting for 4, 0, or 2 points, respectively. The score is totaled and used to grade the impact of tinnitus on daily life on a scale from slight (score of 0–16) to catastrophic (score of 78–100). The survey is self-reported and has very strong internal consistency reliability while also being correlated with other mood scales [36]. 

The TQ is a 52-item questionnaire that assesses the impact of tinnitus across five domains: emotional distress, auditory perceptual difficulties, intrusiveness, sleep disturbances, and somatic complaints. The questions are answered with the response options “true”, “partly true”, or “not true”, which are weighted as 2 points, 1 point, or 0 points, respectively, with a higher score indicating a greater impact [37]. The scale has been shown to be sensitive enough to detect significant changes after treating patients with cognitive behavioral therapy and is best used to separate patients who have tinnitus as their primary complaint from those who report tinnitus as more of a secondary disturbance [38].

The TSS is a 15-item questionnaire which assesses the impact of tinnitus in five domains, including intrusiveness, distress, hearing loss, sleep disturbance, and medication. Responses range in score from 1 (no impact) to 4 (most impact). Each item is weighted from 1 to 3 points. The score is totaled, and a higher score indicates more tinnitus disturbance [39].

The VAS utilizes a 100-point visual scale to quantify the psychometric characteristics of their tinnitus, including loudness (VAS-L), annoyance (VAS-A), distress (VAS-D), and coping (VAS-C), on a 100-point visual analog scale from 0 (no symptoms) and 100 (maximum symptoms) for each scale. It allows the patients to give a detailed description of their tinnitus with relatively few questions and can be translated simply into multiple languages. The correlations between the VAS-L, VAS-A, and VAS-D are the strongest and most reliable, while the VAS-C has a slightly weaker correlation, changing frequently depending on how the patient is coping that day [40].

## 3. Protocols for rTMS and Tinnitus

A review of the most recent trials and meta-analyses reveals that varying protocols are used in determining the current status of efficacy of rTMS treatment for tinnitus. A literature search was conducted utilizing the PubMed and Cochrane Library databases. The terms used in the search were “repetitive transcranial magnetic stimulation” and “tinnitus.” The resulting articles were then further filtered by year (2016–2021) and article type (clinical trial, meta-analysis, randomized-controlled trial (RCT), and systematic review). Of the 30 results in the PubMed search, 16 were included due to the relevance to our review. Others were excluded if they compared rTMS to other non-invasive neuromodulation techniques, targeted other conditions such as epilepsy or depression, or evaluated aspects of rTMS other than efficacy. In the Cochrane Library search, the same parameters were given. Out of the 34 results, three additional RCTs were found beyond what was already established from the PubMed search. Those not relevant to our review were also excluded. Subsequently, four non-randomized studies, 11 RCTs, one systematic review, and three meta-analyses were included in our review. Following the review of the studies, we found discrepancies in the protocol parameters, including the location of treatment, number of sites targeted, number of pulses delivered, frequency of the pulses, duration of treatment and follow-up, and outcome measures. Many aspects of each of the protocols overlapped among the studies. However, even amongst similar protocols, the outcomes varied. A summary of the studies including the frequencies used, the time frame of the trials, the total sessions, the location of the treatments, and the outcome measures are presented in the tables below (Table 1, Table 2 and Table 3).

### 3.1. Frequency, Amount, and Location of Pulses

#### 3.1.1. Frequency

The frequency utilized in rTMS depends on the intended treatment. Low-frequency rTMS has been correlated with a dampening effect on neuroplasticity [9]. Most of the studies assessing rTMS as a treatment for tinnitus that are included in our review used low frequency stimulation aimed at reducing the neural activity in the non-auditory areas related to the pathogenesis of tinnitus. The studies demonstrated a variability in minimum effective stimulation. The level at which overstimulation is reached is also unclear. Of the test parameters examined, the location and combination of locations targeted within rTMS are amongst the most consistent aspects of the protocol. However, there was no consensus on the exact combination of these locations. Given the high variability in testing methods and outcomes, an ideal protocol has yet to be defined.

All four of the studies in Table 1 utilized 1 Hz of frequency for a treatment length of 10 days; however, the total amount of pulses per session varied between 1000 and 2000, with varying results in their respective questionnaires used to evaluate patients post-treatment [13,34,41,42]. Wang et al. [41], the largest experimental study in this group, found that rTMS was successful in nearly half of the patients when treated with 1 Hz directed over the left temporoparietal cortex for 10 days, as evident by a statistically significant improvement in the VAS-L scores in participants used to characterize tinnitus loudness in participants. Negative predictors of treatment success were identified, such as the length of symptoms, presence of hearing loss, and presence of sleep disturbance [41]. In a retrospective study of 199 patients, Yang et al. [13] noted significant results in a total of 62.3% of patients following treatment with 2000 pulses at 1 Hz. They noted the most pronounced improvement, of 82.8%, in the group that only had symptoms for 1 week, compared to 67.2% of patients who had symptoms longer than a year [13]. Kan et al. [42] followed the above-mentioned protocol at the temporoparietal junction (TPJ), noting no significant difference in their patients’ symptoms based on the THI and VAS scores. This lack of improvement may be attributed to the low sample size (11 subjects) or the use of a poor therapeutic target (TPJ). Interestingly, despite not having significant changes in clinical symptoms, anatomical differences in the post-treatment PET scans were identified [42]. These PET scan findings were like those seen in an earlier study by Poeppl et al. [34]. Similarly to the previous two studies mentioned, Poeppl et al. [34] applied rTMS at 1 Hz to the left temporal cortex as well as a high dose of 20 Hz to the left DLPFC. Although they did not have a large number of patients improve clinically, they found that those individuals who responded significantly with a reduction in their TQ score of at least 5 had increased connectivity between the non-auditory brain regions. Because of this, they concluded that there are additional mechanisms and anatomy that are poorly understood in the pathophysiology of tinnitus and should be considered as therapeutic targets [34].

#### 3.1.2. rTMS Pulse Rate

Most RCTs in Table 2 focused on a single-site use of rTMS, targeted at either the DMPFC, AC alone, or temporoparietal region at 1 Hz of stimulation, suggesting the low-frequency model of rTMS as the most widely used protocol [43,45,48,49,50]. Protocols after this point vary quite significantly. In addition to the 1-Hz stimulation, Lehner et al. [44] also used 1000 pulses/day of high-frequency (20 Hz) stimulation applied to the left DLPFC, followed by 1000 pulses/day of low-frequency (1 Hz) stimulation, suggesting the use of a combination of frequencies. Cimenelli et al. [51] utilized a mid-frequency, 10 Hz, directed to the bilateral DMPFC. Kreuzer et al. [52] explored the use of a “standard triple protocol” of 20 Hz stimulation to the DLPFC followed by 1 Hz to the left and right temporoparietal cortex with 1000 pulses compared to the “high-frequency triple” protocol of 20 Hz of the same pulse rate to the same locations. The researchers in the reviewed studies targeted either the temporoparietal region alone, the regions within the frontal lobe alone, or both regions within the same protocol. Noh et al. [45] sought to compare targeting both the AC and DLPFC, as opposed to the studies targeting the DLPFC with the same number of total pulses. Most studies used between 1000 and 3000 total pulses. However, when comparing all studies the highest range is 12,000. Sahlsten et al. [50], who used 4000 pulses, postulated that excessive pulses may have been a factor in producing insignificant changes in the psychometric properties, given that stimulation can lead to the depression or the excitement of the neurons, and that too many pulses may lead to the opposite of the desired effect. 

#### 3.1.3. Location of Treatment

As the neural anatomy for tinnitus is poorly understood, many studies investigating rTMS as a treatment for this symptom may vary on the region(s) of the brain targeted. Broadly speaking, it is mainly the temporal and frontal lobes that have been target either alone or in combination. Seven of the studies included in this review limited their treatment to a single site [12,41,42,43,47,48,49,50], while two studies investigated triple-site therapy [44,52].

Two studies evaluated treating the TPJ as a single site, though neither showed significant clinical findings [42,43]. While Kan et al. [42] showed identifiable differences in the neuronal metabolic activity on the PET scans, Roland et al. [43] did not identify clinically significant functional connectivity changes on resting state functional connectivity MRI (rs-fcMRI). Of note, the two studies discussed treated patients for different lengths of time and with different pulse rates (Table 1 and Table 2). Although further evaluation is needed to form a conclusion, it seems that treating the TPJ as the single treatment site may not lead to a significant symptom improvement, despite the dose and duration of treatment [42,43].

Two studies evaluated the AC as the sole target for rTMS therapy and had opposite results. Cacace et al. [48] found a significant improvement in symptoms, shown by the THQ and magnetic resonance spectroscopy (MRS) in 25 subjects following 5 days of rTMS to the AC. Landgrebe et al. [49] studied a much larger population (163 patients) and were unable to identify any significant difference between the placebo and active rTMS groups. Of note, the patients in the Landgrebe et al. [49] study were treated for a total of 10 days with almost double the number of stimulations as that used in the Cacace study [48]. Sahlsten et al. [50] hypothesized that excessive amounts of pulses can negate any of the positive effects of the treatment, which might have been seen if the data had been collected earlier or their protocol shortened. It is also possible that the significant improvement in symptoms observed by Cacace et al. [48] was due in part to their small cohort of subjects and unclear if similar findings would be observed in a larger population [48,49,50].

Three studies evaluated treatment outcomes with the stimulation of the posterior superior temporal gyrus (STG). James et al. [47] observed significant improvements in tinnitus with 1 Hz stimulation and 10 Hz stimulation, whereas Carter et al. [12] found significant improvements with only 1 Hz stimulation, compared to 10 Hz. Sahlsten et al. [50] studied the effect of 1 Hz stimulation with nearly twice the number of stimulations to the treatment area. They found no difference when comparing the outcomes of their experimental groups to those of their control groups, but both groups reported improved symptoms. This needs to be taken into consideration, as James et al. [47] had no control arm to their study and may have been reporting the placebo effect that Sahlsten et al. [45] found. Carter et al. [12] utilized one group throughout the study, but incorporated sham treatment prior to active treatment [12,47,50].

Lehner et al.’s study [44] was the only one to assess the efficacy of triple-site therapy compared to single-site. They compared the stimulation of the left temporoparietal cortex with the stimulation of both the left DLPFC and bilateral temporoparietal cortices. Patients who received either single-site treatment or triple-site treatment reported a significant benefit in TQ scores, and the magnitude of the improvement was only different at day 90 of the study. The study concluded that multi-site therapy may not be any more beneficial, or harmful, than the treatment of the left temporoparietal cortex alone. However, both groups reported a significant benefit compared to the placebo group, supporting rTMS’ potential use in tinnitus treatment [44].

The remaining studies in Table 1 and Table 2 evaluated dual-site therapy against single-site treatment or placebo. The sites and combinations thereof also varied from study to study. Poeppl et al. [34] and Noh et al. [46] evaluated the stimulation of the TC in addition to the DLPFC. Both reported a significant improvement in symptoms. When Noh et al. [46] compared it to single-site DLPFC alone, they found that dual-site therapy was more effective in reducing tinnitus symptoms at nearly every time point of the study. Both of these studies concluded that the stimulation of non-auditory parts of the brain produces a better therapeutic response based on the VAS and THI scores. Cimenelli et al. [51] compared bilateral DMPFC stimulation to placebo and concluded that their patients had a significant decrease in their symptoms. Although their findings are not generalizable, given their relatively small sample sizes, further studies are necessary to elucidate the efficacy of stimulating these areas of the frontal cortex [34,46,51].

The protocols used in the meta-analyses in Table 3 are consistent with what was found in other studies. The inhibitory frequency of 1 Hz was utilized most frequently with the temporal cortex, temporoparietal area, and regions in the frontal cortex amongst the most commonly targeted regions [53,54,55,56]. Schoisswoh et al. [53] concluded that rTMS therapy is effective in treating tinnitus, that lower-frequency stimulation and a lower dose was associated with significance, and that also treating the prefrontal cortical areas did not significantly change outcomes. Lefebvre-Demers et al. [54] found that there was a significant efficacy in treating tinnitus with rTMS based on a statistically significant decrease in questionnaire scores in their treated patients. They also reported that the treatments targeted at the AC had better outcomes than those targeted at other sites. Liang et al. [56] found that patients had a significant improvement in their tinnitus symptoms at 1 week, 1 month, and 6 months, with a low heterogeneity across studies (I^2^ = 0%, 0%, 21%, respectively) when compared to those who received the placebo treatment. They concluded that rTMS is an efficacious treatment for tinnitus; however, given the dearth of large studies and the lack of standardized protocols, there needs to be further research to verify this treatment. Dong et al. [55] looked at studies that utilized 1 Hz rTMS. Their analysis showed that rTMS had no significant effect, as measured by any questionnaire, in the short or long term. However, they also expressed that rTMS has been shown to be a safe procedure, and therefore further study poses little risk. It was concluded that this analysis was inconsistent with many of the previous studies and may have been limited, given the small sample size and the lack of a standardized protocol. 

### 3.2. Duration of Treatment and Follow-Up

The duration of treatment also varied widely amongst recent studies. The duration of treatment in all the clinical trials in Table 1 was 10 days. The treatment was administered on 5 consecutive days, twice over a 2-week period [13,34,41] or on 10 consecutive days [42]. Yang et al. [13] evaluated patients 3 months following treatment, but there was no additional follow-up period after treatment in any of the other three studies. 

The studies in Table 2 described a larger range of the length of treatments. A 4- and 5-day period of active treatment was utilized by Noh et al. [45,46] and Cacace et al. [48], respectively. Multiple studies utilized a 10-day period of treatment, similar to the trials in Table 1 [44,49,50]. James et al. [47] performed a crossover study where participants received an active treatment of either 1 Hz or 10 Hz for 4 days, and then received sham treatment. This was followed by a 21-day washout period in which they received no treatment, and was then crossed over to a 1-Hz or 10-Hz stimulation, depending on what their initial treatment was, for an additional 4 days. Carter et al. [12] carried out their trial in three different courses of 4 days for each of their treatment periods, leading to 4 days of sham and 8 days of active treatment. Ciminelli et al. [51] and Kreuzer et al. [52] had the longest lengths of treatment, with a 4-week trial and five sessions per week, resulting in 20 sessions total. However, Kreuzer et al. [52] reported that 11 of the 80 patients underwent only 2 weeks of treatment (10 sessions).

Some studies in Table 2 utilized follow-up periods beyond the end of the treatment period to evaluate the efficacy of the rTMS treatment in the long term. The length of the long-term follow-up varied from study to study. The longest follow-up in a study was at day 180. In others, the follow-up was extended for 3 or 4 months [12,44,45,46,50,51,52]. With heterogeneous results within these differing time frames, the most appropriate interval, and therefore the timeline of lasting effects of rTMS, cannot be deduced. 

### 3.3. Primary Baseline and Outcome Measurement Tools

With the variation in patient experience and symptom tolerance, evaluating the outcomes of rTMS has been widely standardized with the use of validated questionnaires that are used in clinical practice to characterize the severity of a patient’s tinnitus. Each study in Table 1 evaluated the tinnitus at baseline and post-treatment with these varying questionnaires, including the TQ [34], the VAS [13,41,42], or the THI [13,42]. Imaging with MRI or PET was used for baseline and outcome evaluation by Poeppl et al. [34] and Kan et al. [42] in order to show functional and metabolic changes in neurons, respectively.

The studies in Table 2 also used a combination of similar techniques for evaluating the effectiveness of rTMS. These included the THI, VAS, TQ, and TSS (Table 2). Imaging techniques such as fMRI and MRS were also used in conjunction with questionnaires to further map neural activity and detect changes in neural metabolism at baseline and the following treatment in some trials [43,48]. Carter et al. [12] also utilized electroencephalography (EEG) to correlate changes in VAS scores and brain wave frequencies.

Similarly to the experimental and RCTs, the meta-analyses in Table 3 also reported the utilization of validated tools such as the THI, VAS, TQ, and TSS across the studies analyzed. No mention of imaging was used throughout the studies. (Table 3).

### 3.4. Efficacy of rTMS in the Treatment of Tinnitus

There have been varying results regarding the efficacy of rTMS for the treatment of tinnitus. Two experimental studies in Table 1 reported significant benefits, suggesting rTMS’s role as a possible treatment modality for tinnitus [34,41]. However, Kan et al. [42] did not observe rTMS to significantly improve tinnitus, attributing the lack of significant results to either the small sample size or limitations associated with targeting the left temporoparietal lobe alone. Yang et al. [13] performed a retrospective study on one group, but reported a significant improvement in a large percentage of patients. With a lack of control groups in the studies in Table 1, it is also necessary to evaluate the efficacy of rTMS in RCT.

Nine out of the eleven RCTs in Table 2 reported significant improvements in tinnitus following treatment with rTMS. Lehner et al. [44] demonstrated significant improvements in tinnitus but with no additional improvements when targeting both the temporoparietal cortex and the DLPFC together, compared to just the left temporoparietal cortex. However, Noh et al. [46] did achieve significance in their trial when comparing targeting both the AC and DLPFC and targeting the AC alone. With the largest trial to date determining the efficacy of rTMS to the AC alone, Landgrebe et al. [49] did not observe significant results. Roland et al. [43] also targeted only the temporoparietal junction and did not report significant results. This suggests the possibility that the efficacy of multi-site therapy depends on the other parameters of the protocols [44,46]. 

Three out of the four reviews in Table 3 observed significant improvements in tinnitus with rTMS treatment compared to the outcomes observed in those receiving sham treatment. However, the heterogeneity among the current treatment protocols may limit the understanding of the effect of rTMS on tinnitus [53,54,56]. Finally, Dong et al. [55] suspected that their small sample size was not large enough to demonstrate a significant benefit. 

## 4. Conclusions and Future Directions

With the ability to impact both neural connections between regions of the brain and the gene expression of particular neurons, rTMS can influence neuroplasticity on both the macro- and the microscopic level. A review of the current literature revealed significant improvements in the perceptual properties of tinnitus, including intensity, annoyance, and distress, as well as its impact on the quality of life, following treatment with rTMS. A significant obstacle to the clinical application of rTMS in the treatment of tinnitus is a lack of standardized treatment parameters. The protocols used in the studies reviewed in this analysis vary most widely in terms of the dose of pulses, duration of treatment, and interval of follow-up. Though it remains unclear which specific testing parameters, and in what combination, would result in the greatest improvement in tinnitus perception and reaction, the studies examined in this review suggest that rTMS may be an effective treatment modality for tinnitus. Further evaluation could help define a standardized clinical protocol and establish a path to the clinical application of rTMS in the treatment of tinnitus.

## Figures and Tables

**Table 1:** Non-randomized studies: Summary of characteristics, protocols, results, and conclusions in 4 studies utilizing rTMS on patients with tinnitus without randomization.

Author	Year	Subject Number	Primary Baseline Evaluation	rTMS Protocol (Session Number, Frequency, Amount of Stimuli)	Location of Treatment	Primary Evaluation of Outcome	Results	Conclusions
Wang et al. [41]	2016	-289 patients with chronic tinnitus -30 healthy control	-Tinnitus loudness determined by visual analog scale (VAS) -Hearing level with audiometer -Tinnitus loudness evaluated with TinniTest audiometer	-Underwent repetitive magnetic transcranial stimulation (rTMS) over the left temporoparietal cortex region. -Stimuli consisted of 1000 stimuli at 1 hertz (Hz) daily and 110% of the motor cortex threshold for 5 consecutive days per week for 2 weeks (10 sessions total). -Control received same treatment	-Left temporoparietal cortex	-VAS score after last treatment	-rTMS showed an effect in 138 of the patients (47.8%) and no effect in 151 patients (52.2%) in the active group. -VAS average prior was 5.5 and 2.7 after -Significant tinnitus suppression found in patients with shorter tinnitus duration, normal hearing, and without sleep disturbance.	-rTMS resulted in a significant reduction in tinnitus loudness -Study states imaging would help determine the best site of treatment
Poeppl et al. [34]	2018	-60 patients with chronic tinnitus -0 control	-MRI immediately before treatment -Tinnitus Questionnaire (TQ)	-Underwent 10 consecutive days with 10 sessions -Patients received rTMS of the left DLPFC (40 trains with 50 stimuli; 25 s intertrain interval; 20 Hz; 110% resting motor threshold (RMT)), followed by low-frequency rTMS (2000 Stimuli; 1 Hz; 110% RMT) of the left temporal cortex.	-Left dorsolateral prefrontal cortex (DLPFC) and left temporal cortex (TC)	-Magnetic resonance imaging (MRI) after last treatment -Responders classified as scoring 5 points fewer on tinnitus questionnaire	-Assessed for longitudinal gray matter changes and structural connectivity -Longitudinal mesoscopic gray matter changes of DLPFC, left operculo-insular, and right inferior temporal Cortex (ITC) in responders (*n* = 22) but not in non-responders (*n* = 38) -Increased connectivity in DLPFC–insula and insula–ITC in responders. Weak DLPFC–insula connectivity and no insula–ITC connectivity in non-responders.	-Results support the role of non-auditory brain regions in tinnitus and as possible therapeutic targets in rTMS.
Kan et al. [42]	2019	-11 patients with idiopathic tinnitus -11 healthy controls	-Tinnitus handicap inventory (THI) and VAS -Positron emission tomography (PET) scans before treatment for regions of increased activity in idiopathic tinnitus compared to controls	-1000 TMS pulses at a frequency of 1 Hz for a total of 30 min for 10 consecutive days, once a day	-Left temporoparietal cortex	-Tinnitus handicap inventory (THI) score -VAS score -PET scan -All after last treatment	-No significant statistical difference before and after treatment regarding THI score (t = 1.019, *p* = 0.342 > 0.05) and VAS (*t* = 0.00, *p* = 1.0 > 0.05). -Posttreatment PET scan showed increased activities in the right parahippocampal gyrus, right superior temporal gyrus, right superior frontal gyrus, anterior insula, left inferior parietal lobule, and left precentral gyrus. -Decreased activities were noted in the left postcentral gyrus and left inferior temporal gyrus (ITG)	-Noted limitations by small sample size -Left temporoparietal cortex alone may not be sufficient
Yang et al. [13]	2021	-199 patients with tinnitus identified in a retrospective review	-THI and VAS	-Each patient underwent 10 sessions, 5 sessions a week for 2 weeks -2000 stimuli per session of 1 Hz	-Left temporal cortex and left prefrontal cortex	-At 3-month follow-up THI and VAS reevaluated. -A reduction in THI score by more than 6 points and VAS by 1 or more from the baseline result was considered effective	-62.3% of all patients responded based on THI scores and 66.3% based on VAS score. -Patients with shorter duration of illness (1 week) responded the best to treatment with a rate of 82.8% versus 57.6%, 53.5%, and 67.2% for patients of 1-week to 1-month, 1-month to 1-year, and over 1-year duration	-rTMS is effective in treating tinnitus, but the efficacy is dependent on the duration of symptoms prior to treatment

**Table 2:** Randomized-controlled trials: Summary of characteristics protocols, results, and conclusions in 11 RCTs utilizing rTMS on patients with tinnitus.

Author	Year	Subjects per Group and Blinding	Primary Baseline Evaluation	rTMS Protocol Group 1 (Session Number, Frequency, Amount of Stimuli)	Protocol Group 2	Primary Evaluation of Outcomes	Results	Conclusion
Roland et al. [43]	2016	-Group 1 (experimental): 16 patients with nonpulsatile tinnitus -Group 2 (Sham): 14 patients with nonpulsatile tinnitus -Double-blinded	-Resting state functional connectivity MRI (rs-fcMRI) -THI	-Group 1: Active treatment was delivered at 1 Hz at 110% of RMT at the temporoparietal junction for 42.5 min (2500 stimuli) with interval stimulation for 2 or 4 weeks.	-Group 2 (sham): same protocol with placebo rTMS	-rs-fcMRI following treatment for 2 or 4 weeks -THI following treatment	-No statistically significant changes found between pre and post intervention in both the rs-fcMRI and THI	-Concluded both a lack of symptom change and neural connectivity changes -Suggest they may not have had sufficient stimulation to the area of interest or should consider non-auditory brain regions associated with tinnitus
Lehner et al. [44]	2016	-Group 1: 24 patients with tinnitus (Single site) -Group 2: 25 patients with tinnitus (triple site) -Group 3: 25 patients with tinnitus (placebo) -Double-blinded	-8 various tinnitus questionnaires	-Group 1 (single site): 3000 pulses/day of the left temporoparietal cortex with low-frequency (1 Hz) rTMS of the left temporoparietal cortex -Group 2 (triple site): 1000 pulses/day of high-frequency 20 Hz stimulation of the left DLPFC, followed by 1000 pulses/day of low-frequency (1 Hz) to both the left and right temporoparietal cortex (3000 pulses total) -Ten sessions total for each group	Group 3 (placebo): sham coil was localized at the auditory cortex by using a PET-guided neuronavigation system.	-8 tinnitus questionnaires on the last day of treatment (day 12), day 90 and day 180 following treatment	-Both the single site and triple site showed statistically significant reductions in tinnitus severity, but the difference between the two is not significant besides at day 90.	-Study did not find significant differences between one or multi-site rTMS treatment -More work needed on exact protocols for a more effective and individualized treatment
Noh et al. [45]	2017	-Group 1: 9 patients with tinnitus -Group 2: 13 patients with tinnitus -Blinding not possible	-THI score -VAS score	-Group 1: Auditory cortex (AC) and frontal cortex (FC) determined by 10–20 EEG system -rTMS was administered at a frequency of 1 Hz with an intensity of 110% RMT -40 s on and 20 s off -Total of 12,000 pulses: 2000 pulses over the AC, and 1000 pulses over the FC daily for 4 days.	-Group 2: coil navigated to the primary AC and FC by a MRI neuronavigation system -Same rTMS treatment as group 1	-THI weeks 1, 4, and 8 after baseline -VAS at weeks 1, 4, 8, and 12 after baseline	-Both groups had a significant reduction in THI scores -Group 1 effect lasted 8 weeks and group 2 effect lasted 4 weeks, but the differences were not statistically different -VAS score reduction not statistically significant in group 1 but statistically significant in group 2 up to 12 weeks post treatment -ΔVAS between the groups was not statistically significant	-Localizing technique for treatment target not a crucial factor in the rTMS outcome of the same locations
Noh et al. [46]	2017	-Group 1: 9 patients with chronic tinnitus (dual-site) -Group 2: 8 patients with chronic tinnitus (single-site) -Double blinded	-THI score -VAS score -State-Trait Anxiety Inventory (STAI) -Beck’s Depression Inventory (BDI) -Pittsburgh Sleep Quality Index (PSQI)	Group 1: Low frequency (1 Hz) treatments with 2000 pulses applied to the AC and 1000 pulses applied to the DLPFC for 4 days (total of 12,000 pulses)	Group 2: Low frequency (1 Hz) treatments with 3000 pulses applied to the DLPFC for 4 days (total of 12,000 pulses).	-THI, VAS, STAI, PSQI at weeks 1, 2, 4, and 12 after treatment	-Group 1 showed significant reductions in THI and VAS scores at all weeks of evaluation, whereas group 2 did not -Group 1 showed significant improvements in STAI at 12 weeks, and PSQI scores at 4 weeks. -Group 2 showed a significant improvement only in STAI at 12 weeks.	-Targeting both the AC and DLPFC had better outcomes in all areas of evaluation in comparison to just targeting the DLPFC -Non-auditory cortex stimulation only is not sufficient to impact tinnitus
James et al. [47]	2017	-12 total participants in a crossover study -Half started at 1 Hz, the other half at 10 Hz, with a sham in between crossover -Double-blinded	-fMRI -Visual Analog Rating (VAR)	-Both groups received: Sham, 1 Hz, and 10 Hz for four sessions per arm, 1800 pulses per session, delivered at 110% of RMT over the posterior superior temporal gyrus (STG)	-Groups crossed over with a 21-day washout period	-fMRI after final treatment -VAR after final treatment	-Both 1 Hz and 10 Hz rTMS stimulation showed changes in tinnitus awareness from baseline compared to sham -All three measures of the VAR (awareness, loudness, and annoyance) were improved with 1 Hz. -Higher DLPFC activity at baseline may predict a poorer response to rTMS	-Using 1 Hz and 10 Hz can lead to similar results, even though they lead to inverse effects on neural excitability -The role of DLPFC plays a role in tinnitus and rTMS responsiveness
Cacace et al. [48]	2017	-25 total participants with chronic tinnitus -Single-blinded crossover	-THI for inclusion -Audiogram -Tinnitus Handicap Questionnaire (THQ) -Metabolite levels using magnetic resonance spectroscopy (MRS)	-Active rTMS: 1 Hz and at a power setting of 110% of RMT over the left AC -20-min sessions with a total of 1200 stimuli -Participants received 5 days of active rTMS and then 5 days of sham rTMS stimulation, sequentially	-Sham treatment with same time frame	-Audiogram at day 5 -THQ at day 5 -MRS at day 5	-Significant decrease in the loudness of tinnitus -Significant reduction in THQ score -Down regulation in the glutamate (excitatory) seen in the left and not the right hemisphere	-Perceptual, psychoacoustic, and neurochemical analysis of rTMS treatment showed improvement in tinnitus
Landgrebe et al. [49]	2017	-163 patients with chronic tinnitus -Group 1: 75 patients (experimental) -Group 2: 78 patients (sham) -Patient and rater blinded	-Tinnitus Questionnaire (TQ) -THI -Tinnitus Severity Scale (TSS)	-Group 1: 10 sessions active 1-Hz-rTMS (2000 stimuli, 110% RMT) to the left temporal cortex (AC).	-Group 2: Sham rTMS with the same time frame	-TQ, THI, TSS at day 12	-No statistically significant difference in outcome measures between the active and the sham group	-No effect found in the largest trial testing rTMS of the left AC alone -Larger trials of other protocols should be carried out
Sahlsten et al. [50]	2017	-39 patients with chronic tinnitus -Group 1: 19 patients (experimental) -Group 2: 20 patients (placebo) -Single-blinded	-THI -VAS -Audiogram	-Group 1: 10 sessions over 2 weeks of 4000 pulses at 1 Hz to the left superior temporal gyrus (STG) at 100% of RMT	-Group 2: Placebo rTMS	-THI, VAS after 10 days, at 1 month and at 3 months post treatment	-Significant reduction in THI score in both groups but not between groups -Significant decrease in mean intensity, annoyance and distress VAS scores at 3 months post-treatment but not significant over time -No changes in hearing in both groups	-Improvement in both VAS and THI in the whole study group but not between groups -May be attributed to too many pulses or placebo effect -Best protocol of rTMS remains uncertain
Ciminelli et al. [51]	2020	-Group 1 (Experimental): 15 patients with tinnitus -Group 2 (Sham): 14 patients with tinnitus -Single-blinded	-THI score -VAS score -Tinnitus loudness	-Group 1 (experimental group): Each session of 10 Hz stimulation applied 3000 pulses to each DMPFC for 15 min each side (6000 pulses total). -5 s on and 10 s off were used, for a total time of 30 min -Treatment was 5 times a week for 4 weeks (20 total sessions)	-Group 2 (sham) received the same protocol but with a placebo coil -Coil produced the same sound and sensation	-THI and VAS score at weeks 1, 2, and 4 of treatment and 16 weeks after baseline -Tinnitus loudness following treatment	-A significant difference of 11.53 in THI (95% confidence interval [CI]: −23.12 to 0.06; p = 0.05) -VAS difference of 0.80 not statistically significant (95% CI, −2.21 to 0.61) -Tinnitus loudness score reduction of 4.46 dB was borderline significant (95% CI: −9.60 to 0.68 dB; *p* = 0.09).	-Results show a benefit in 2/3 parameters used to evaluate tinnitus
Kreuzer et al. [52]	2021	-Exploratory open label study -Randomized, parallel-group design with 80 patients -32 received “standard triple protocol” (Group 1) -38 received “high-frequency triple protocol” (Group 2)	-Primary baseline: TQ	-“Standard triple” protocol: 20 Hz stimulation of the DLPFC followed by 1 Hz to the left and right temporoparietal cortex with 1000 stimuli for 4 weeks - Total of 3000 stimuli per session -6 patients were treated for only 2 weeks	-“High-frequency triple” protocol: 20 Hz to the left DLPFC and the left and right temporoparietal junction area with 1000 stimuli for 4 weeks -Total of 3000 stimuli per session -5 patients were treated for only 2 weeks	-Primary outcome: TQ assessed at baseline, week 2, week 4, week 12	-Change in TQ from baseline to week 12 was significant (P = 0.016). -No significant interaction effect between measurement time point (2 vs. 4 weeks) and group (standard vs. high-frequency)	-Due to the pilot nature of the study, clinical relevance remains unknown -4 weeks is a feasible treatment time, but not superior to 2 weeks -High frequency not superior or inferior to the standard therapy
Carter et al. [12]	2021	-Double-blinded, sham-controlled -19 patients in crossover study -All patients received Sham (Group 1) -All patients received treatment (Group 2)	-Electro-encephalography (EEG) -VAS and line mark (LM) rating of loudness, annoyance, awareness -THQ	-Group 1: All participants received sham rTMS first -Three 4-day courses of participants received 1800 pulses at a 110% motor threshold targeted over the posterior, superior temporal gyrus.	-Group 2: Participants were randomized to either the 1 Hz and 10 Hz and then crossed over to second frequency after completing the first -Three 4-day courses of rTMS participants received 1800 pulses targeted over the posterior, superior temporal gyrus	-VAR/LM and EEG evaluation at baseline, the end of each treatment week and 2 months following treatment	-No significant change in VAS compared to before and immediately following treatment, during sham, or active 10-Hz treatment -1-Hz treatment led to a significant decrease in both the LM and VAS awareness ratings at days 1–3 with loudness (*p* = 0.0447), annoyance (*p* = 0.0195), and awareness (*p* = 0.0430) -No changes in any EEG frequency band between baseline and sham -EEG after 1 Hz: significant increase in beta and delta coherence -EEG after 10 Hz: increase in theta and beta coherence	-No immediate effect of rTMS on tinnitus during a single rTMS session -1 Hz was associated with a decrease in tinnitus awareness and was associated with an increase in beta coherence -EEG changes noted in treatment responders but absent in non-responders and sham treatment -Beta coherence is a possible biomarker of the rTMS effect

**Table 3:** **Meta-analyses and systematic reviews:** Summary of characteristics, results, and conclusions of 1 systematic review and 3 meta-analyses of RCTs regarding the efficacy of rTMS for the treatment of Tinnitus.

Author	Year	Number of Studies Analyzed and Years of Interest	Location Target(s)	Neuromodulation Frequencies	Main Outcome Assessment	Heterogeneity (I^2^ Analysis)	Results	Conclusions
Schoisswoh et al. [53]	2019	-55 significant study arms from 2005 to 2017 -18 insignificant study arms from 2007 to 2017 -Randomized controlled trials	-Temporal cortex (*n* = 32,9) -Temporoparietal cortex (*n* = 23, 9) -Prefrontal in addition to AC (*n* = 9,7)	-Inhibitory: 1 Hz, cTBS (*n* = 49, 18) -Excitatory: 10 Hz, 25 Hz (*n* = 6, 0)	-Chi-squared analysis of reported significant and not significant results of study arms	-Not applicable, only a systematic review performed	-Higher efficacy in active rTMS compared to sham rTMS -Lower stimulation intensity associated with significance -Lower number of pulses increased significance -Adding prefrontal cortical areas did not contribute to significance	-Meta-analysis would have given less of a dichotomized result -There are many factors that go into rTMS efficacy in treating tinnitus -The prefrontal cortex may not be significant due to the addition of more pulses -rTMS protocols need to be more standardized for a definitive analysis
Lefebvre-Demers et al. [54]	2020	-28 studies from 2004 to 2019 -Randomized controlled trials	-Auditory cortex (*n* = 16) -Temporoparietal area (*n* = 17) -DLPFC and left AC (*n* = 3) - DLPFC and both AC (*n* = 1) -Frontal cortex (*n* = 1)	-1 Hz (*n* =20) -10 Hz (*n* =1), -50 Hz cTBS (*n* = 4) -27,12 MHz (n 1⁄4 1) -≥1 frequency (20 Hz and 1 Hz, *n* = 1; 25 Hz and 1 Hz, *n* = 1)	-THI (*n* = 20) -Tinnitus Questionnaire (TQ) (*n* = 6) -Tinnitus Functional Index (TFI) (*n* = 1) -Tinnitus Severity Index (TSI) (*n* = 1)	-moderate total heterogeneity (54.9%)	-Sample size: 34 ± 29 participants -Tinnitus outcomes: Pre- to post-rTMS Hedges g-value of 0.45 (CI = 0.66; 0.24; *p* < 0.0001), showing a moderate effect -Active rTMS showed a statistically significant mean change in questionnaire scores of 7.60 -Location: rTMS targeting the AC significantly reduced symptoms compared to other sites	-rTMS is an effective treatment option for tinnitus based on the effect on standardized questionnaires
Dong et al. [55]	2020	-10 studies from 2010 to 2019 -Randomized controlled trials	-Temporal cortex/auditory cortex only (*n* = 7) -Temporoparietal cortex only (*n* = 2) -Temporal with the frontal regions (*n* = 1)	-1 Hz with 100% or 110% RMT with varying pulses of 1000, 1020, 1500, 2000, 3000, and 4000)	-THI only (*n* = 4) -TQ only (*n* = 1) -VAS only (*n* = 1) -THI and TQ (*n* = 1) -THI and VAS (*n* = 1) -THI, TQ, and VAS (*n* = 2)	-No heterogeneity	-A pooled analysis showed that active rTMS had no significant effect on THI scores compared with sham in the short term, medium term, or long term -A pooled analysis showed no significant effect of active rTMS on the loudness assessed by VAS in the short term, medium term, or long term -A pooled analysis showed no significant improvement of the severity assessed by TQ in the short term, medium term, or long term	-The review showed no significant improvement of tinnitus symptoms following rTMS treatment compared to sham -Inconsistent with previous studies and may be limited to a small sample size
Liang et al. [56]	2020	-29 studies from 2010 to 2019 -Randomized controlled trials	-Auditory cortex (*n* = 27) -Motor cortex (*n* = 1) -Not specified (*n* = 1)	-1 Hz most frequently used (93.1%)	-THI and TQ scores at 1 week, 2 weeks, 1 month, and 6 months post treatment	-0% at 1 week -0% at 1 month -21% at 6 months	-Significant difference in THI scores at 1 week, 1 month, and 6 months compared to sham -Significant difference in TQ scores 1-week post treatment compared to sham	-Efficacy of active rTMS compared to sham proven in the analysis -More studies needed for further confirmation

## Data Availability

Not applicable.
